# Supplementary material for: SWELL1 regulates skeletal muscle cell size, intracellular signaling, adiposity and glucose metabolism
Source: eLife. 2020 Sep 15;9:e58941. doi: 10.7554/eLife.58941 (PMC7541086; doi:10.7554/eLife.58941)
Supplement: Supplementary file 3. [file elife-58941-supp3.docx]

|  |  |  | |  |
| --- | --- | --- | --- | --- |
|  | **Male** | | **Female** | |
|  | **WT** | **KO** | **WT** | **KO** |
| **Total:** | 18 | 19 | 20 | 15 |
| **%** | ***21.9*** | ***23.1*** | ***24.3*** | ***18.2*** |

**Supplementary Table 3: Genotypes from *Myf5^Cre^/Lrrc8a^fl/fl^ breeding***

**WT: *Lrrc8a^fl/fl^***; **KO:** ***Myf5^Cre^/Lrrc8a^fl/fl^* (*Lrrc8a* KO**)
